# Impact of caffeine on myocardial perfusion reserve assessed by semiquantitative adenosine stress perfusion cardiovascular magnetic resonance

**DOI:** 10.1186/s12968-019-0542-7

**Published:** 2019-06-24

**Authors:** Andreas Seitz, Philipp Kaesemann, Maria Chatzitofi, Stephanie Löbig, Gloria Tauscher, Raffi Bekeredjian, Udo Sechtem, Heiko Mahrholdt, Simon Greulich

**Affiliations:** 1 Department of Cardiology, Robert Bosch Medical Center, Auerbachstraße 110, 70376 Stuttgart, Germany; 2 Department of Cardiology and Angiology, University Hospital Tübingen, Tübingen, Germany

**Keywords:** Caffeine, Ischemia, Adenosine stress CMR, Myocardial perfusion reserve, MPRI, Splenic switch-off

## Abstract

**Background:**

Adenosine is used in stress perfusion cardiac imaging to reveal myocardial ischemia by its vasodilator effects. Caffeine is a competitive antagonist of adenosine. However, previous studies reported inconsistent results about the influence of caffeine on adenosine's vasodilator effect. This study assessed the impact of caffeine on the myocardial perfusion reserve index (MPRI) using adenosine stress cardiovascular magnetic resonance imaging (CMR). Moreover, we sought to evaluate if the splenic switch-off sign might be indicative of prior caffeine consumption.

**Methods:**

Semiquantitative perfusion analysis was performed in 25 patients who underwent: 1) caffeine-naïve adenosine stress CMR demonstrating myocardial ischemia and, 2) repeat adenosine stress CMR after intake of caffeine. MPRI (global; remote and ischemic segments), and splenic perfusion ratio (SPR) were assessed and compared between both exams.

**Results:**

Global MPRI after caffeine was lower vs. caffeine-naïve conditions (1.09 ± 0.19 vs. 1.24 ± 0.19; *p* <  0.01). MPRI in remote myocardium decreased by caffeine (1.24 ± 0.19 vs. 1.49 ± 0.19; *p* <  0.001) whereas MPRI in ischemic segments (0.89 ± 0.18 vs. 0.95 ± 0.23; *p* = 0.23) was similar, resulting in a lower MPRI ratio (=remote/ischemic segments) after caffeine consumption vs. caffeine-naïve conditions (1.41 ± 0.19 vs. 1.64 ± 0.35, *p* = 0.01). The SPR was unaffected by caffeine (SPR 0.38 ± 0.19 vs. 0.38 ± 0.18; *p* = 0.92).

**Conclusion:**

Caffeine consumption prior to adenosine stress CMR results in a lower global MPRI, which is driven by the decreased MPRI in remote myocardium and underlines the need of abstinence from caffeine. The splenic switch-off sign is not affected by prior caffeine intake.

## Background

Adenosine stress cardiovascular magnetic resonance (CMR) is a routine diagnostic tool in the assessment of ischemia in patients with known or suspected coronary artery disease (CAD) [1]. In clinical routine, physicians often encounter patients who report recent caffeine consumption despite instructions to avoid caffeine 24 h before the exam. Caffeine is a nonselective competitive inhibitor of adenosine2A-receptors, which might attenuate the vasodilator effect of adenosine, and increases sympathetic activity yielding to capillary de-recruitment, leading to decreased myocardial perfusion reserve [2, 3]. However, studies investigating the effect of caffeine on adenosine-induced hyperemia reported inconsistent results: We and others have previously reported that caffeine consumption prior to adenosine stress perfusion CMR led to a reduction of ischemic burden on qualitative assessment [4–7]. Other groups, however, did not observe significant impact of caffeine on stress perfusion defect size [8, 9]. Data on (semi-)quantitative assessment of myocardial perfusion are scarce, a submaximal hyperemic effect of adenosine after caffeine consumption is supposed [10], and seems reasonable. As a result, there continues to be debate in the clinical routine, if a scheduled adenosine stress CMR should be cancelled, or performed in patients presenting with caffeine consumption during the last 12–24 h before the exam [11].

In the present study, we sought to investigate in patients with substantial myocardial ischemia under caffeine-naïve conditions: 1) the effect of a defined caffeine intake on adenosine stress CMR using semiquantitative perfusion analysis of ischemic and remote myocardial segments, and 2) if the splenic switch-off sign, which has recently been suggested as indicator for adequate hyperemia [12], is sensitive to prior caffeine consumption.

## Methods

### Patient population

This prospective study applied comprehensive semiquantitative analysis of myocardial perfusion to assess the effect of caffeine consumption on adenosine stress perfusion CMR. In addition, the effect of caffeine on the splenic switch-off sign, which has recently been suggested as indicator for adequate adenosine stress, was investigated using visual and semiquantitative assessment. The cohort of this study was part of a previously reported trial, in which we found that caffeine consumption led to a slight reduction of perfusion defect size determined by visual assessment of adenosine stress CMR. The detailed design and main results of this previous trial have recently been published [4]. Briefly, between May 2014 and September 2015, 25 patients were enrolled in this substudy. Inclusion criteria were: 1) the evidence of significant myocardial ischemia (≥2 segments according to the 16-segments American Heart Association (AHA) model [13]) on clinically indicated adenosine stress perfusion CMR under caffeine-naïve conditions and 2) the visibility of the spleen on perfusion images. Five patients of the original cohort were excluded: 4 patients as the spleen was not visible on perfusion images and one patient as rest perfusion image quality at the caffeine -naïve scan was impaired due to extrasystole, potentially hampering semiquantitative perfusion analysis. Participants underwent repeat adenosine stress perfusion CMR after prior intake of 2 cups of coffee (~ 200 mg of caffeine) [14]. Caffeine intake was scheduled one hour before the stress perfusion CMR in order to ensure maximum serum caffeine levels [15, 16]. Blood samples were taken from the participants at each CMR exam, and serum caffeine levels were determined using an immune-assay technique (Bioscientia, Ingelheim, Germany). According to the internal standards at our institution, patients were instructed to refrain from caffeine as well as all anti-anginal medications within 24 h before both CMR examinations. Between the first and the repeat CMR exam, no coronary intervention was performed. However, all patients underwent invasive coronary angiography, which confirmed significant coronary artery stenosis (≥70% diameter stenosis). Written informed consent was obtained from all patients and the study protocol has been approved by the ethics committee of the University of Tübingen, Germany.

### CMR imaging

Adenosine stress perfusion CMR was performed according to a standardized protocol, which has previously been described [17]. Briefly, patients were scanned in the supine position in a 1.5 T scanner (MAGNETOM Aera, Siemens Healthineers, Erlangen, Germany) using electrocardiogram (ECG)-gating sequences. Balanced steady-state free-precession cine images were acquired in short- and long-axis views to assess the size and function of the left ventricle (LV). Subsequently, 3 short-axis slices representing the basal, mid-ventricular and apical LV myocardium were selected for perfusion imaging. Hyperemia was induced by intravenous administration of adenosine (140 μg/kg/min) under continuous heart rate and blood pressure monitoring. After at least 3 min of adenosine injection, a gadolinium-based contrast agent (0.07 mmol/kg gadopentetate) was injected at 4 ml/s and first-pass perfusion was imaged using a saturation-recovery, gradient-echo TurboFLASH (fast low-angle shot) sequence. After a 15 min break, rest perfusion without adenosine was performed. Late gadolinium enhancement (LGE) was performed 5 min after rest perfusion using a segmented inversion-recovery sequence.

### CMR analysis

**Fig. 1:**
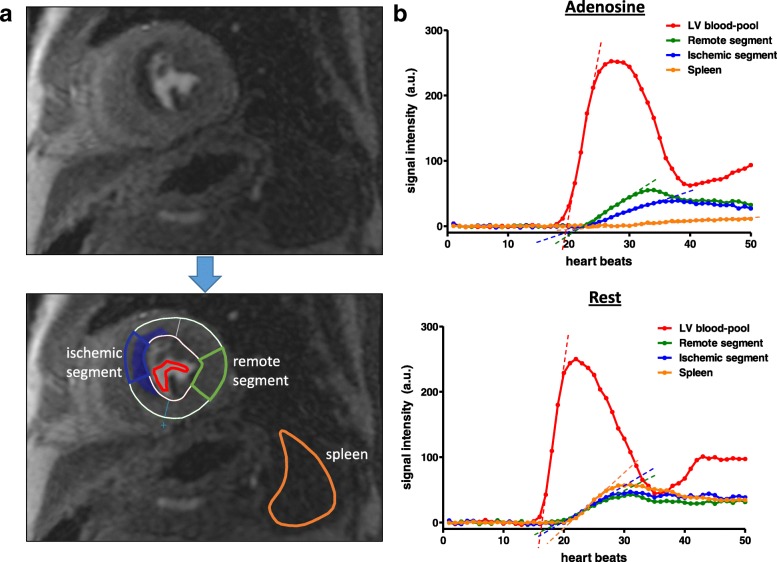
**a**) Myocardial and splenic perfusion was assessed by semiquantitative analysis. **b**) Signal intensity-over-time curves were generated for the LV blood-pool (red curve), 16 myocardial (American Heart Association (AHA)-) segments representative for the left ventricular (LV) myocardium (blue curve = ischemic segment; green curve = remote segment) and the spleen (orange curve). Maximum upslope (dashed lines) of each curve was determined by linear fitting. Myocardial and splenic relative upslope (RU) was calculated by dividing the maximum upslope of the myocardial/splenic signal intensity curve through the maximum upslope of the LV-blood-pool curve. Myocardial perfusion reserve index (MPRI) and splenic perfusion reserve (SPR) represent the ratio of stress and rest perfusion

Analyses of LV systolic function and LGE were performed by two experienced observers (S.G., P.K.) blinded to the patient’s clinical status, caffeine status and other test results (e.g. coronary angiography) as previously described [4]. Stress and rest perfusion images were evaluated using qualitative and semiquantitative assessment by two experienced observers (A.S., S.G.). A perfusion defect was defined as subendocardial or transmural area of hypoperfusion on adenosine stress perfusion lasting for at least 2 heartbeats, which was reversible during rest perfusion [18]. Signal intensity-over-time curves were generated for all myocardial segments during stress and rest perfusion, respectively, using dedicated software (QMASS, Medis, Leiden, the Netherlands), Fig. 1. The maximum upslope for each segment was determined by applying a linear fit of 5 data points of the signal intensity curves. Moreover, maximum upslope was determined for the LV blood-pool using a linear fit of 3 data points. A third region of interest was placed in the spleen in order to determine signal intensity changes and the maximum upslope of the splenic perfusion. Relative upslope (RU) was calculated for each myocardial segment as the ratio of the maximum myocardial upslope divided by the maximum upslope of the LV blood-pool to account for differences in the arterial input function, as previously described [19]. Myocardial perfusion reserve index (MPRI) was calculated by dividing hyperemic RU through the RU at rest. Moreover, splenic RU at rest and stress and the splenic perfusion ratio (SPR) were calculated accordingly. Finally, each myocardial segment was graded as: 1) ischemic, 2) ischemic in part, or 3) remote, by both observers (A.S., S.G.) in consensus. For further analysis, ischemic segments were compared to remote segments, while “ischemic in part” segments were excluded from the segment-based analysis to minimize potential influence of differences in the segmentation of the LV myocardium. Global MPRI was calculated for all 16 segments irrespective of ischemic involvement [13]. The MPRI ratio was defined as MPRI of remote myocardium divided by MPRI of ischemic segments, and served as an indicator for contrast intensity between ischemic and remote myocardial perfusion areas, with higher values allowing better discrimination of remote from ischemic regions by visual assessment.

### Statistical analysis

Baseline characteristics and CMR parameters are expressed as absolute numbers and percentages to describe the study population. Continuous variables are expressed as means with standard deviation (SD) or medians with interquartile range (IQR). Comparisons between groups were made using the student’s *t* test or the Mann-Whitney U test, as appropriate. A two-tailed *P*-value of < 0.05 was considered statistically significant. All statistical analyses were performed using GraphPad Prism (version 5.01, GraphPad Software, San Diego, California USA).

## Results

### Patient characteristics

Overall, 25 patients (84% male; median age 69 years) were included in the final cohort, including 48% who had previously known CAD. Patients were habitual caffeine consumers with a mean daily consumption of 3 cups of caffeinated coffee or 4 cups of caffeinated tea, respectively. The majority of patients suffered from angina (56%) or dyspnea (36%); 8% were asymptomatic. Caffeine serum levels at the initial caffeine-naïve CMR were below the detection limit (< 1 mg/L), whereas caffeine levels at the second exam (after defined intake of 2 cups of coffee) were increased (4.6 ± 2.3 mg/L), Table 1.

**Table 1:** Baseline and CMR characteristics

	Study cohort *n* = 25
General	
Age, years	69 (62–75)
Male sex, n (%)	21 (84)
Hypertension, n (%)	20 (80)
Diabetes mellitus, n (%)	9 (36)
Family history of CAD, n (%)	11 (44)
Current smoking, n (%)	2 (8)
Symptoms	
None	2 (8)
Angina	14 (56)
Typical angina	12 (48)
CCS 1	–
CCS 2	7 (28)
CCS 3 and 4	5 (20)
Atypical angina	2 (8)
Dyspnea	9 (36)
NYHA I	–
NYHA II	8 (32)
NYHA III/IV	1 (4)
Known CAD	12 (48)
Caffeine consumption and serum levels	
Coffee, cups	3 (2–4)
Tea, cups	4 (2–4)
Caffeine level baseline CMR, mg/L	< 1
Caffeine level follow-up CMR, mg/L	4.6 ± 2.3
CMR routine parameters	
LVEF, %	64 ± 6
LVEDVi, mL/m^2^	130 ± 32
LVESVi, mL/m^2^	49 ± 19
IVS thickness, mm	13 ± 3
LA, cm^2^	21 ± 4
Ischemic segments (16-segments model)	7.4 ± 3.2
Days between baseline and follow-up CMR	12 (3–14)

Data are n (%), mean ± SD or median (IQR)

CAD, coronary artery disease; CCS, Canadian Cardiovascular Society class; NYHA, New York Heart Association class; CMR, cardiac magnetic resonance imaging; LVEF, left ventricular ejection fraction; LVEDVi, left ventricular end-diastolic volume index; LVESVi, left ventricular end-systolic volume index; IVS, interventricular septum; LA, left atrium

### Baseline CMR characteristics

Patients demonstrated a preserved LV ejection fraction of 64 ± 6%, and a normal LV end-diastolic volume index, Table 1. The median time period between the initial (caffeine-naïve) and the repeat (after defined caffeine intake) adenosine stress CMR was 12 (3–14) days. Hemodynamics (blood pressure, heart rate) during stress perfusion and rest perfusion were similar at both CMR exams, Table 3.

### Semiquantitative perfusion analysis - regional and global

**Fig. 2:**
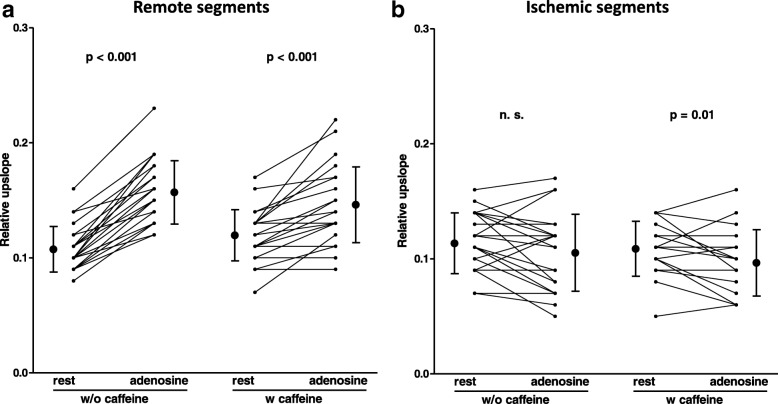
RU of (**a**) remote and (**b**) ischemic myocardial segments at rest and during adenosine-induced hyperemia (=stress). RU of remote myocardial segments was significantly increased by adenosine, irrespective of caffeine intake. Ischemic segment RU was not significantly influenced by adenosine on caffeine-naïve exams, while it was slightly reduced by adenosine after caffeine intake

**Fig. 3:**
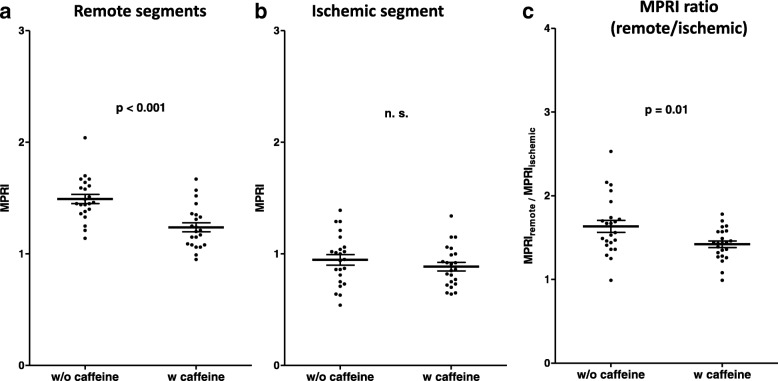
**a** MPRI of remote myocardial segments was significantly higher without caffeine compared to measurements after caffeine intake. **b** In contrast, caffeine had no significant effect on MPRI of ischemic segments. **c** Subsequently, the ratio of remote and ischemic segment MPRI was increased without caffeine, indicative of a better contrast ratio between ischemic and remote myocardial perfusion, facilitating the visual assessment of ischemia

Myocardial perfusion was assessed by semiquantitative analysis as described above, Fig. 1. The RU of remote myocardial segments on the caffeine-naïve stress CMR under adenosine was higher compared to the RU at rest (15.7 ± 2.8 vs. 10.7 ± 2.0, *p* <  0.001), Fig. 2a, Table 2. After caffeine consumption, remote myocardial segments showed increased RU at rest (12.0 ± 2.2 vs. 10.7 ± 2.0, *p* <  0.01) and decreased RU under adenosine (14.6 ± 3.3 vs. 15.7 ± 2.8, *p* <  0.05) compared to the caffeine-naïve CMR exam, Fig. 2a, Table 2. Subsequently, MPRI of remote myocardium under caffeine-naïve conditions was significantly higher than after defined caffeine intake (1.49 ± 0.19 vs. 1.24 ± 0.19, *p* <  0.001), Fig. 3a**,** Table 2.

**Table 2:** Semiquantitative analysis of ischemic and remote segment perfusion

	baseline (w/o caffeine) *n* = 25		p	follow-up (w/ caffeine) *n* = 25		p	p (baseline vs. follow-up)	
	ischemic	remote		ischemic	remote		ischemic	remote
RU rest	11.4 ± 2.6	10.7 ± 2.0	0.40	10.9 ± 2.4	12.0 ± 2.2	0.32	0.27	**< 0.01**
RU adenosine	10.5 ± 3.3	15.7 ± 2.8	**< 0.001**	9.7 ± 2.9	14.6 ± 3.3	**< 0.001**	0.13	**< 0.05**
RU_rest_/RU_adenosine_ = MPRI	0.95 ± 0.23	1.49 ± 0.19	**< 0.001**	0.89 ± 0.18	1.24 ± 0.19	**< 0.001**	0.23	**< 0.001**
MPRI_remote_/MPRI_ischemic_ = MPRI ratio	1.64 ± 0.35			1.41 ± 0.19			**0.01**	

Data are mean ± SD

RU, relative upslope; MPRI, myocardial perfusion reserve index

< 0.05 entries are in bold

Among ischemic segments, the resting RU after caffeine consumption was similar compared to the resting RU on the caffeine-naïve CMR (10.9 ± 2.4 vs. 11.4 ± 2.6, *p* = 0.27), Fig. 2b**,** Table 2. After caffeine intake, RU of ischemic segments was lower during adenosine compared to rest (9.7 ± 2.9 vs. 10.9 ± 2.4, *p* = 0.01), Fig. 2b**,** Table 2. However, MPRI of ischemic segments did not change by caffeine consumption compared to the caffeine-naïve exam (0.89 ± 0.18 vs. 0.95 ± 0.23, *p* = 0.23), Fig. 3b**,** Table 2.

We calculated the ratio of remote and ischemic segment MPRI as an indicator for the contrast intensity, allowing better discrimination between remote and ischemic perfusion areas. This MPRI ratio was significantly higher in the caffeine-naïve adenosine stress perfusion CMR (1.64 ± 0.35 vs. 1.41 ± 0.19, *p* = 0.01), Fig. 3c**,** Table 2.

Including all 16 myocardial (AHA-) segments of the LV irrespective of the presence of ischemia, RU both at rest and at stress were not significantly influenced by the intake of caffeine. However, global MPRI was higher under caffeine-naïve conditions (1.24 ± 0.19 vs. 1.09 ± 0.19, *p* <  0.01), Table 3. No correlation was found between caffeine levels and the magnitude of MPRI reduction after caffeine consumption (r = 0.13, *p* = 0.52).

**Table 3:** Semiquantitative analysis of myocardial and splenic perfusion

	baseline (w/o caffeine) *n* = 25	follow-up (w/ caffeine) *n* = 25	p
**Hemodynamic status during CMR**			
HR at rest, /min	67 ± 9	71 ± 11	0.27
Systolic BP at rest, mmHg	151 ± 22	152 ± 26	0.78
Diastolic BP at rest, mmHg	88 ± 13	87 ± 9	0.76
HR during adenosine, /min	86 ± 12	85 ± 8	0.71
Systolic BP during adenosine, mmHg	147 ± 20	150 ± 23	0.38
Diastolic BP during adenosine, mmHg	88 ± 12	87 ± 8	0.79
**Global myocardium (all 16 segments)**			
RU rest	11.1 ± 1.9	11.4 ± 2.0	0.52
RU adenosine	13.3 ± 2.3	12.5 ± 2.8	0.11
MPRI	1.24 ± 0.19	1.09 ± 0.19	**< 0.01**
**Spleen**			
RU rest	15.6 ± 5.1	16.2 ± 6.4	0.73
RU adenosine	5.6 ± 3.3	5.9 ± 3.0	0.66
SPR	0.38 ± 0.19	0.38 ± 0.18	0.92

Data are mean ± SD

RU, relative upslope; MPRI, myocardial perfusion reserve index; SPR, splenic perfusion ratio; HR, heart rate; BP, blood-pressure

### Assessment of the splenic switch-off sign

**Fig. 4:**
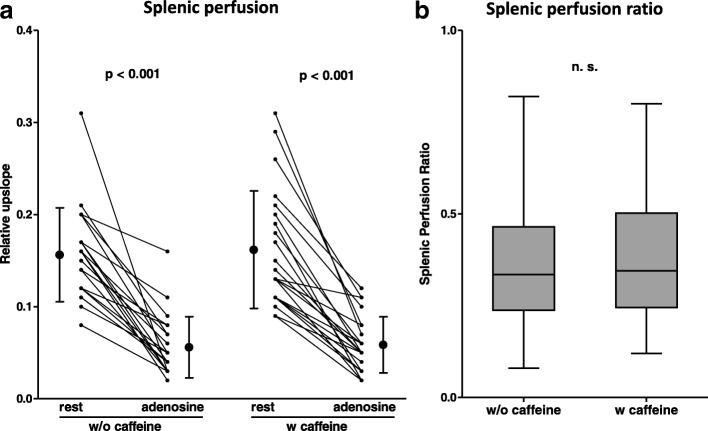
**a** Splenic perfusion (RU) was reduced (“switch-off”) during adenosine vs. rest perfusion on all stress CMR exams, indicating adequate adenosine-induced hyperemia, irrespective of the intake of caffeine. **b** Likewise, the splenic perfusion ratio was not influenced by caffeine. Thus, the splenic switch-off did not allow for detection of prior caffeine consumption in this study

By visual assessment, the splenic switch-off sign on stress perfusion images could be observed in all adenosine stress CMR exams, independent of prior caffeine intake. Likewise, on semiquantitative analysis, rest perfusion of the spleen was higher compared to adenosine stress, again irrespective of the caffeine status (with caffeine: 16.2 ± 6.4 vs. 5.9 ± 3.0, *p* <  0.001; without caffeine: 15.6 ± 5.1 vs. 5.6 ± 3.3, p <  0.001), Fig. 4a**,** Table 3. Moreover, the SPR, which was calculated as the splenic equivalent to the MPRI, was similar in both initial and repeat exams (0.38 ± 0.19 vs. 0.38 ± 0.18, *p* = 0.92), Fig. 4b**,** Table 3**.**

## Discussion

To our knowledge, this is the first study applying semiquantitative analysis of adenosine stress perfusion CMR to investigate the impact of caffeine on myocardial perfusion in patients with substantial myocardial ischemia (7.4 ± 3.2 segments) under caffeine-naïve conditions. The main findings of this study are as follows: 1) Compared to the caffeine-naïve adenosine CMR, defined intake of 2 cups of coffee (equivalent to ~ 200 mg caffeine) prior to adenosine stress CMR resulted in reduced global MPRI results, which were driven by a decreased MPRI of the remote myocardium, whereas the MPRI of ischemic myocardium was unaffected; 2) Despite significant reduction of the MPRI, prior intake of 200 mg caffeine did not outweigh the hyperemic effect of adenosine on remote myocardial perfusion, and 3) Neither visual nor semiquantitative assessment of the splenic switch-off sign could reveal caffeine intake prior to the CMR exam.

### Patient characteristics

At the time of the initial CMR, serum caffeine levels of all patients were below the detection limit (< 1 mg/L), Table 1. At the repeat adenosine stress CMR, caffeine levels had increased to a range indicating a substantial effect from caffeine on the vasodilator effect of adenosine as shown by previous studies in which myocardial blood flow was assessed by positron emission tomography (PET) or invasive fractional flow reserve (FFR), respectively [10, 20, 21].

### Semiquantitative perfusion analysis - remote myocardium

Previous studies have investigated the impact of caffeine on adenosine stress perfusion imaging using single photon emission computed tomography (SPECT), CMR or PET [6–10]. Despite overall inconsistent results about the impact of caffeine on adenosine stress exams, most groups found a reduced ischemic myocardial burden in some degree, if stress perfusion imaging was performed after caffeine consumption using the standard dose of adenosine (140 μg/kg/min) [4–7, 10]. However, in the majority of studies, ischemic burden was defined rather by the size of perfusion defects and number of ischemic segments than by (semi-)quantitative assessment of myocardial perfusion. In the present study, we performed semiquantitative analysis of adenosine stress perfusion CMR and found that MPRI of remote myocardium was lower on stress CMR after caffeine consumption compared to the caffeine-naïve CMR, Fig. 3a, Table 2. This decrease of MPRI by caffeine corroborates previous studies using myocardial blood flow assessment using cardiac PET stress imaging [10, 20]: Investigating the effect of caffeine on myocardial blood flow and myocardial perfusion reserve in 10 healthy controls, Kubo et al. found that caffeine (mean plasma caffeine level of 3.1 ± 1.6 mg/L) resulted in a decrease of myocardial perfusion reserve from 5.2 ± 1.6 to 2.4 ± 0.9 [10]. Another study found an inverse relation of myocardial perfusion reserve and caffeine serum levels using dipyridamole PET in healthy controls [20]. To our knowledge, no previous study has been published using semiquantitative analysis of adenosine stress perfusion CMR to assess the impact of caffeine on myocardial perfusion reserve in patients with substantial myocardial ischemia under caffeine-naïve conditions. In two recently published CMR studies applying native T1 mapping at stress and rest, myocardial blood volume under adenosine was decreased in subjects with self-reported consumption of 1–2 cups of coffee < 4 h prior to the exam compared to caffeine-naïve patients [5, 22].

In remote segments, RU after caffeine intake was enhanced in rest perfusion but decreased under adenosine compared to the corresponding caffeine-naïve CMR, Fig. 2a, Table 2**,** which is in line with previous studies [10]. Namdar et al. found in a group of healthy controls a significant increase of resting myocardial blood flow by caffeine at hypoxia. At normoxia, they observed an + 11.4% increase in rest perfusion after consumption of 200 mg caffeine (equivalent to our dosage) compared to caffeine-naïve conditions, however this was not statistically significant [3]. In this study, we found an almost similar increase of remote myocardial RU at rest (+ 12.1%), which was statistically significant most likely due to the larger cohort size (*n* = 25 vs. *n* = 10), Table 2. One explanation for increased myocardial rest perfusion induced by caffeine could be explained by its stimulating effect on catecholamine release [3, 14].

### Semiquantitative perfusion analysis - ischemic myocardium

While MPRI of remote myocardial segments was decreased by caffeine, no significant impact of caffeine on MPRI in ischemic segments could be detected, Fig. 3a-b**,** Table 2. Of note, previously mentioned studies performed quantification of myocardial perfusion (or myocardial blood volume) assessment by PET (or CMR) in subjects without coronary artery stenosis. Conversely, we focused exclusively on patients with significant obstructive CAD. Hence, there is no literature yet for the finding that caffeine demonstrated no significant effect on the MPRI of ischemic segments. Interestingly, under caffeine-naïve conditions, RU under adenosine was slightly decreased compared to the RU at rest, Fig. 2b**,** Table 2. This finding might be attributed to the impaired vasodilatory capacity of the diseased coronary artery and the previously described “coronary steal effect” due to the coronary artery stenosis [23].

### Semiquantitative perfusion analysis - entire myocardium and MPRI index

As a result of the decreased MPRI of remote myocardial segments, global MPRI was significantly lower after caffeine intake compared to caffeine-naïve exams, Table 3. Recent studies suggest a high diagnostic accuracy of MPRI assessment not only for the presence of epicardial coronary stenosis, but also for the work-up of suspected coronary microvascular dysfunction [24]. Cut-off values for MPRI and for absolute myocardial blood flow determined by adenosine stress perfusion CMR have been proposed for the diagnosis of obstructive epicardial or microvascular CAD [24]. We recently demonstrated that caffeine intake prior to adenosine stress perfusion CMR reduced ischemic burden, but did not impact sensitivity of visual assessment (no conversion from positive to negative exam) [4]. However, the present data demonstrate a reduction of MPRI by caffeine, which may result in false-positive exams. However, further studies are needed to test this hypothesis including patients with a lower ischemic burden (e.g. 2 ischemic segments) and control patients without ischemia to assess precisely the impact of caffeine on sensitivity and specificity. Nevertheless, despite the increasing use of quantitative analysis in recent studies, adenosine stress CMR in daily clinical practice is still predominantly assessed qualitatively by visual aspects only. For this purpose, distinct contrast intensity between ischemic and remote myocardium on stress perfusion images is essential. Furthermore, perfusion defect size on adenosine stress imaging had been demonstrated to be smaller after caffeine compared to caffeine-naïve conditions, hampering the visual assessment additionally [4–7]. In the present study, we calculated the ratio of remote and ischemic segment MPRI as an indicator for the contrast intensity between ischemic and remote myocardial perfusion on visual assessment. This ratio was significantly increased in the caffeine-naïve adenosine stress perfusion CMR (1.64 ± 0.35 vs. 1.41 ± 0.19, *p* = 0.01), suggesting an improved contrast intensity for the visual analysis, facilitating detection and assessment of the true size of stress perfusion defects, Fig. 3c, Table 2. Detecting the true extent of myocardial ischemia is not only of diagnostic but also of prognostic value [25]. Hence, in order to preserve the high diagnostic accuracy of adenosine stress perfusion CMR [1], we still suggest to refrain from caffeine intake prior adenosine stress CMR exams.

### Splenic switch-off sign

The splenic switch-off sign had initially been suggested as an observational tool to assess adequate hyperemia during adenosine stress perfusion CMR [12, 26]. Some studies have applied quantitative measures of the splenic switch-off sign using T1 mapping or semiquantitative splenic perfusion analysis [22, 27, 28]. In this study, splenic switch-off was observed in all CMR studies under adenosine by both qualitative and semiquantitative perfusion analysis irrespective of the presence of caffeine, Fig. 4 and Table 3. Thus, neither visual nor semiquantitative assessment of the splenic switch-off sign allowed detection of prior caffeine intake. This finding is in accordance with a study, which found no differences regarding the response of splenic T1 values to adenosine comparing patients with coffee-intake < 4 h before the CMR to caffeine-naïve patients [22]. The self-controlled design of the present study adds stronger evidence to this observation.

### Limitations

There are several limitations to this study. Due to the single-center design, we cannot exclude center-specific bias. Furthermore, semiquantitative perfusion analysis is known to be influenced by non-linearity of signal intensity and gadolinium concentration [29]. This limitation can be largely overcome by fully quantitative perfusion analysis, however, this requires the use of a dual-bolus or a dual-sequence approach, which was both not available in our patients [30, 31]. Moreover, MPRI values in this study were lower compared with studies from other groups [19, 32]. This is explained, at least in part, by the use of software which is known to produce generally lower MPRI values compared to competitor software [33]. Another reason might be the study cohort consisting exclusively of patients with significant coronary stenosis leading to significant myocardial ischemia. Therefore, we presume a high prevalence of concomitant coronary microvascular disease in these patients. However, invasive testing of coronary flow reserve or microvascular resistance using intracoronary pressure and flow measurements, which would have helped to elucidate this aspect, was not part of the study design. Another limitation of our study is the inter-study variability for semiquantitative/quantitative adenosine stress CMR perfusion estimation, for which controversial results have been reported by previous studies [34–36]. Regarding our standardized protocol and the short inter-study gap (12 [3 to 14] days), which is in the range of prior studies reporting good reproducibility [34, 35], we assume low inter-scan variability in this study.

## Conclusion

Among patients with substantial myocardial ischemia at adenosine stress perfusion CMR under caffeine-naïve conditions, caffeine consumption prior to CMR led to a reduced MPRI of the global LV myocardium, primarily driven by a decreased MPRI of remote segments. The contrast intensity between ischemic and remote regions, crucial for the qualitative visual approach to detect potential ischemia, was enhanced without caffeine. Moreover, neither visual nor semiquantitative assessment of the splenic switch-off sign allowed detection of prior caffeine intake. Therefore, we suggest caffeine abstinence prior to adenosine stress perfusion CMR to facilitate confident diagnosis of myocardial ischemia.

## Data Availability

All patients´ files and results are stored in the Robert Bosch Medical Center, Stuttgart, Germany.

## Abbreviations

AHA: American Heart Association
CAD: Coronary artery disease
CMR: Cardiovascular magnetic resonance
ECG: Electrocardiogram
EDVi: End-diastolic volume index
EF: Ejection fraction
FFR: Fractional flow reserve
LGE: Late gadolinium enhancement
LV: Left ventricle/left ventricular
MPRI: Myocardial perfusion reserve index
PET: Positron emission tomography
RU: Relative upslope
SPECT: Single photon emission computed tomography
SPR: Splenic perfusion ratio
